# Dim artificial light at night alters immediate early gene expression throughout the avian brain

**DOI:** 10.3389/fnins.2023.1194996

**Published:** 2023-07-04

**Authors:** Cassandra K. Hui, Nadya Chen, Arunima Chakraborty, Valentina Alaasam, Simon Pieraut, Jenny Q. Ouyang

**Affiliations:** Department of Biology, University of Nevada, Reno, Reno, NV, United States

**Keywords:** cFos, ZENK, zebra finch, *Taeniopygia guttata*, light pollution

## Abstract

Artificial light at night (ALAN) is a pervasive pollutant that alters physiology and behavior. However, the underlying mechanisms triggering these alterations are unknown, as previous work shows that dim levels of ALAN may have a masking effect, bypassing the central clock. Light stimulates neuronal activity in numerous brain regions which could in turn activate downstream effectors regulating physiological response. In the present study, taking advantage of immediate early gene (IEG) expression as a proxy for neuronal activity, we determined the brain regions activated in response to ALAN. We exposed zebra finches to dim ALAN (1.5 lux) and analyzed 24 regions throughout the brain. We found that the overall expression of two different IEGs, cFos and ZENK, in birds exposed to ALAN were significantly different from birds inactive at night. Additionally, we found that ALAN-exposed birds had significantly different IEG expression from birds inactive at night and active during the day in several brain areas associated with vision, movement, learning and memory, pain processing, and hormone regulation. These results give insight into the mechanistic pathways responding to ALAN that underlie downstream, well-documented behavioral and physiological changes.

## Introduction

A continued rise in global urbanization also increases artificial light at night (ALAN), with light pollution now recognized as a disruptive pollutant (Dominoni and Nelson, 2018). ALAN, even at dim levels, disrupts physiological and behavioral processes (Ouyang et al., 2018). However, these changes appear uncoupled from canonical circadian genes, which synchronize behavior and physiology to the natural photoperiod (Spoelstra et al., 2018; Alaasam et al., 2021), but see (Dominoni et al., 2020). Therefore, how dim ALAN affects neuronal activity to disrupt downstream physiological and behavioral processes remains unknown. This knowledge gap hinders our ability to predict and ameliorate responses to light pollution.

As ALAN disrupts hormone regulation, immune function, and nighttime activity, its effect could be linked to many corresponding brain regions (Alaasam et al., 2018; Mishra et al., 2019), especially if central circadian pacemakers are not disrupted. For example, ALAN disrupts melatonin and diurnal corticosterone production (Mishra et al., 2019), which are produced by the adrenal and pineal glands and directly regulate the hypothalamus, septum, and hippocampus (Chabot et al., 1998; El-Sherif et al., 2003; Kus et al., 2013; Zhang et al., 2017). ALAN also disrupts immune gene expression, neuronal survival, and plasticity in the hippocampus and caudal nidopallium (Mishra et al., 2019; Taufique et al., 2019; Moaraf et al., 2020; Namgyal et al., 2020). Lastly, ALAN recruits new neurons to the medial striatum, theorized to replace dying neurons (Moaraf et al., 2021).

Neuronal activity induces immediate early gene (IEG) expression for new protein synthesis (Greenberg et al., 1986; Flavell and Greenberg, 2008). Therefore, IEGs indicate neuronal activation by associating firing with gene expression and have successfully been used to map neuronal pathways (Guzowski et al., 2005; Feenders et al., 2008). IEGs, such as cFos and ZENK, have been shown to respond to different stimuli. cFos expression is stimulated by cAMP and calcium, and ZENK expression by injury, stress, etc. (Morgan and Curran, 1988; Sagar et al., 1988; O’Donovan et al., 1999). Using both IEGs can generate a holistic, detailed map of brain activity for a more representative analysis (Feenders et al., 2008; Nordmann et al., 2020).

We analyzed ALAN’s impact on IEG expression throughout the whole brain of Zebra finches (*Taeniopygia guttata*), an excellent diurnal model organism, as they translate external light similarly to most vertebrates (Durstewitz et al., 1999; Nakane and Yoshimura, 2014). Since ALAN initiates nighttime activity, we predicted activation in the visual and motor pathways, but that these areas would be similar to birds awake during the day. We also predicted, based on previous research, activation in areas involved in learning and memory, particularly the hippocampus, caudal nidopallium, and striatum (Taufique et al., 2019; Moaraf et al., 2021). We found that ALAN significantly altered IEG expression of cFos and ZENK in the hyperpallium, mesopallium, nidopallium, para-hippocampalis, striatum, entopallium, arcopallium, hippocampus, and septum compared to day and/or night birds.

## Methods

### Experimental design

Thirteen male zebra finches (~100 days old) were kept in outdoor aviaries at the University of Nevada, Reno with no previous exposure to ALAN. When they were ~ 140 days old, we moved them to individually housed indoor 47 cm × 31 cm × 36 cm cages and entrained them to 12 h light and 12 h dark (12 L,12D) for 4 weeks. For daylight, we used 1.4-Watt 5,000 K light emitting diode (LED) rated at 95 Lumens lights at 0:00 (zeitgeber time (ZT) 0) and lights off at 12:00 (ZT 12). Birds were given food and water *ad libitum*. Each cage contained a mechanized perch that relayed hop activity to MATLAB every minute. Cages had individual light-occlusion shades and constant white noise in the background to limit visual and acoustic cues.

We video recorded 30 min of behavior 90 min before perfusion and activity via automated perches (Alaasam et al., 2018). An observer blind to the treatments determined time spent eating/drinking, grooming, hopping, or no movement for the video recordings. We conducted a power analysis based on previously collected behavior data from control and ALAN exposed birds and determined that at least 3 birds were needed per treatment group (Power = 0.8, *α* = 0.05, effect size = 2, number of groups = 3).

Birds were randomly assigned to one of 3 conditions: control night (12 h light: 12 h dark; 12 L:12D sacrificed at dark night: ZT 14, *n* = 4), control day (12 L:12D sacrificed at day: ZT 10, *n* = 4), and experimental ALAN (12 h light: 12 h dim light; 12 L:12Ldim sacrificed at night with artificial light: ZT 14, *n* = 5). We chose the control day timepoint as close as possible to the night timepoints to be certain in capturing awake birds but also avoiding larger differences in circadian activity. As determined by One-Way ANOVA, groups did not differ in initial mass (*p* = 0.62). After the 4-week entertainment period, we sacrificed the control night group during the dark period (ZT 14) and the control day group during the light period (ZT 10). We sacrificed individuals in the ALAN group 2 h after the bird’s 1^st^ exposure to ALAN (ZT 14), to obtain peak protein expression and avoid overlap from the light period. ALAN was standardized to around 1.5 lux ±0.01 from a 20 cm × 1.5 cm 5000 K broad spectrum LED strip. This was done with an Extech Easyview Digital Light Meter (model EA13) and lux was calculated using a mean measurement at perch height and two opposing base corners. For a full-spectrum description of the lights, please see (Alaasam et al., 2021).

### Immunohistochemistry

We anesthetized birds with 0.1 ml of anesthesia made from 30 mg Ketamine HCl, 105 mg Xylazine, and 8.25 ml saline. After no response to a hard toe pinch, weight was taken, and we perfused birds with 1X PBS for 5 min and 4% paraformaldehyde in 1X PBS (PFA) for 13 min. Brains were removed, left in 4% PFA for 24 h, switched to a 15% sucrose solution for 4–12 h, followed by a 30% sucrose solution overnight, and then flash frozen with powdered dry ice and stored in −80°C until slicing.

We cut the left hemisphere of the brain sagittally at 45 μm thickness in six series. Series 1 was stained for imaging (total slices analyzed: ALAN = 90, control day = 67, control night = 67) and 2–6 were stored in cryoprotectant (3.3% sucrose, 0.01% Polyvinyl-pyrrolidone (PVP-40), 30% ethylene glycol in 0.1 M Phosphate Buffer) in −80°C.

We incubated brain slices in a blocking solution (4% BSA, 0.4% triton, 0.05% Na-Azide in 1x PBS) for 3 h and then with primary antibody diluted in blocking solution (c-Fos anti-rabbit polyclonal from ABCAM (ab190289) diluted 1:1000, ZENK anti-mouse monoclonal received from Dr. Keays’ lab (Nordmann et al., 2020) diluted 1:300) in 4°C for ~46 h. We washed slices 3 times in 1X PBS for 25 min each at room temp and incubated overnight at 4° C with secondary antibodies (anti-rabbit 488 (from ABCAM ab150081) diluted 1:1000 and anti-mouse 594 (from ABCAM ab150116) diluted 1:1000), protected from light. We then incubated slices with DAPI for 15–25 min at room temperature and washed them in 1X PBS 3 times. Slices were mounted with antifade mounting medium (VECTASHIELD®) on slides. We imaged tile scans of full slices within 1 week of mounting on a Leica TCS SP8 confocal microscope.

### Statistical analyses

We analyzed images on ImageJ. We determined brain regions using anatomical locations with DAPI staining and a reference atlas from zebrafinchatlas.org. Cells were determined positive for cFos or ZENK if they were three times the mean brightness and overlapped with DAPI. We divided the number of positive IEG cells by the total DAPI cell count to determine expression percentage in representative areas measured over several slices.

We performed statistical analyses in R, version 4.1.2 (R Development Core Team, 2011). We ran generalized linear mixed-effect models to assess if IEG expression levels were affected by the treatment group as a fixed effect (*lme4* package). Slice number and bird ID were included as random effects. We used a Kruskal-Wallis test to analyze the interaction of behaviors and treatment groups. We ran a correlation matrix for all brain regions in each treatment for both cFos and ZENK (Supplementary Figure S1).

### Ethics statement

All procedures were conducted in accordance with the National Institute of Health Ethical Use of Animals and approved by the University of Nevada, Reno Institutional Animal Care and Use Committee.

## Results

### Activity

**Figure 1:**
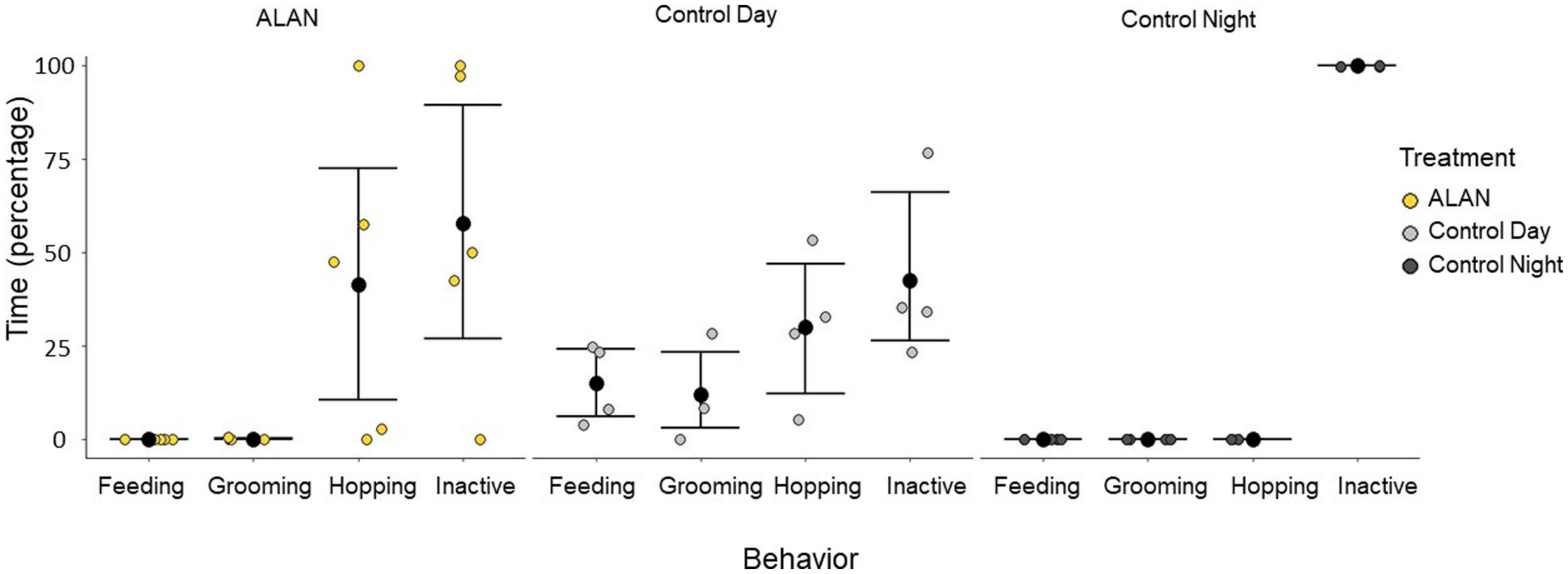
Types of behavior 75 to 105 min before perfusion for birds exposed to ALAN and control birds collected during the day and night. A 30-min window of time 90 min before perfusion (75 to 105 min) was analyzed and broken down into four different behaviors: feeding (eating or drinking), grooming, hopping, and inactive. Shown are means ± 1 SE.

There was a significant difference in the interaction between behavior and treatment group (Kruskal-Wallis test: Grooming: chi-squared = 6.22, *p* = 0.05, Feeding: chi-squared = 11.51, *p* < 0.01, Hopping: chi-squared = 6.45, *p* = 0.04, Inactive: chi-squared = 7.20, *p* = 0.03). Hop activity measured via perch recordings also showed that birds had significantly lower nocturnal activity (control night) than daytime activity (control day) and nocturnal activity under ALAN (Kruskal-Wallis test: chi-squared = 6.47, *p* = 0.04, Figure 1).

### IEG expression

**Figure 2:**
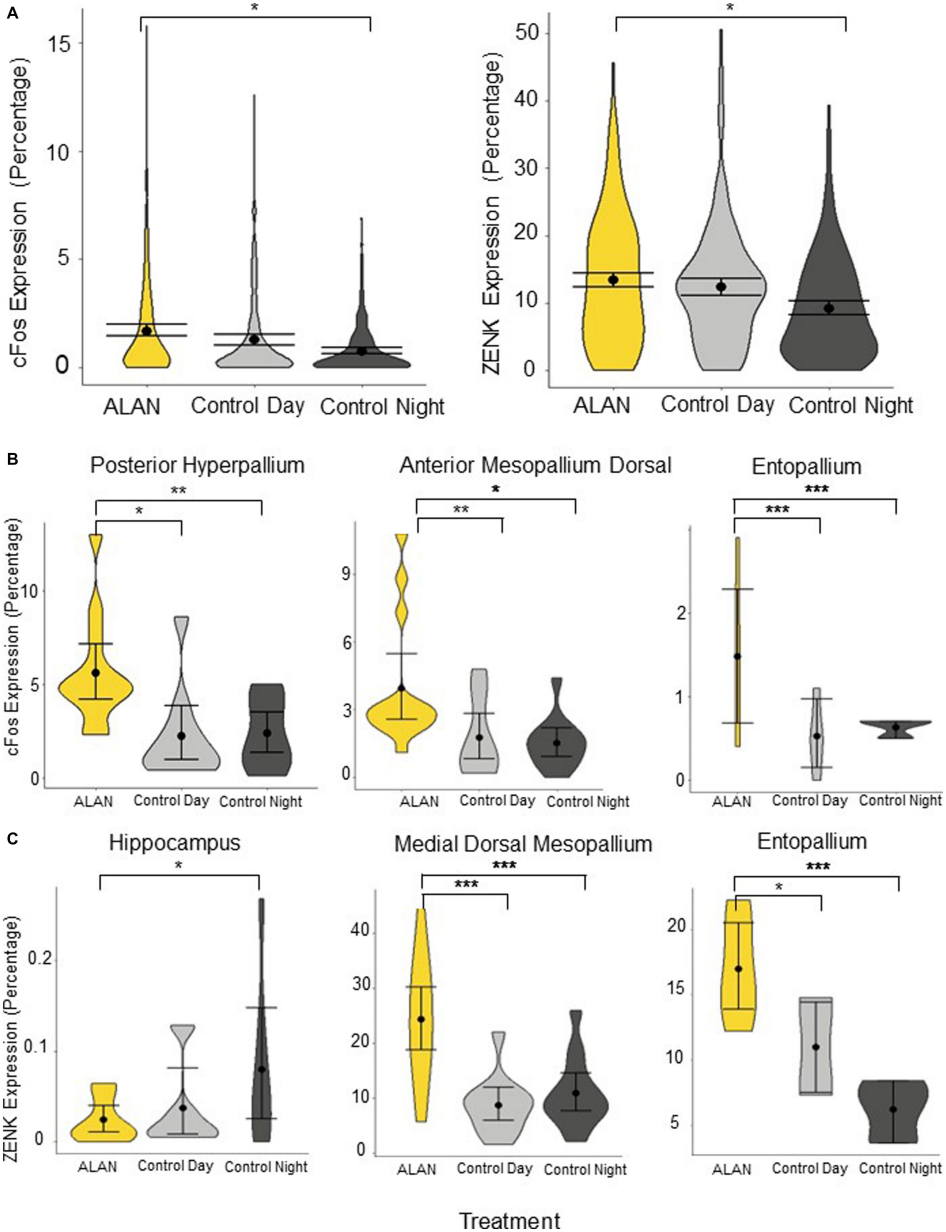
Immediate early gene expression of cFos and ZENK throughout the brain for birds exposed to ALAN, and control birds collected during subjective day and night. **(A)** Total cFos and ZENK expression, shown in percentages. Expression is significantly higher in the ALAN treatment group compared to the night controls but not the day controls. **(B)** cFos expression (percentage) comparing birds exposed to ALAN to control day and control night groups in three brain regions: posterior hyperpallium, anterior mesopallium dorsal, and entopallium. **(C)** ZENK expression (percentage) comparing birds exposed to ALAN to control day and control night groups in three brain regions: hippocampus, medial dorsal mesopallium, and entopallium. Displayed are representative brain regions from *a priori* hypotheses, please see Supplementary Figures S2, S3 for all brain regions. Shown are means ±1 SE. Significance stars: *p < 0.05, **p < 0.01, ***p < 0.001.

The ALAN group was significantly different from the control night (cFos *p* = 0.027, ZENK: *p* = 0.037) but not the control day (cFos: *p* = 0.17, ZENK: *p* = 0.66) when all 24 brain regions were analyzed together (Figure 2A). We broke down the analysis by looking into two major pathways—motor and visual—as well as additional areas. There was no significant difference between cFos and ZENK expression between the ALAN group and either control in all combined areas analyzed in the motor pathway (cFos-Day: *p* = 0.40, cFos-Night: *p* = 0.14, ZENK-Day: *p* = 0.72, ZENK-Night: *p* = 0.14). Similarly, we saw no significant difference for all areas analyzed in the visual pathway (cFos-Day: *p* = 0.08, cFos-Night: *p* = 0.07, ZENK-Day: *p* = 0.61, ZENK-Night: *p* = 0.11). However, individual areas in both pathways were significantly different (Table 1; Supplementary Figures S2, S3).

**Table 1:** Analysis of IEG expression, arranged alphabetically by region, for individual brain regions comparing control night and control day groups against birds exposed to ALAN.

Brain area	Pathway	cFos significance	cFos Z value	ZENK significance	ZENK Z value
Anterior Hyperpallium	Motor	ALAN = Night (*p* = 0.076)	−1.65	ALAN = Night (*p* = 0.189)	−1.31
		ALAN = Day (*p* = 0.099)	−1.78	ALAN = Day (*p* = 0.752)	−0.32
Anterior Mesopallium Dorsal	Motor	ALAN ≠ Night**(*p* = 0.039)**	−2.07	ALAN ≠ Night**(*p* = 0.017)**	−2.40
		ALAN ≠ Day**(*p* = 0.008)**	−2.64	ALAN = Day (*p* = 0.145)	−1.46
Anterior Mesopallium Ventral	Motor	ALAN ≠ Night**(*p* = 0.018)**	−2.36	ALAN = Night (*p* = 0.154)	−1.43
		ALAN = Day (*p* = 0.898)	0.13	ALAN = Day (*p* = 0.858)	−0.18
Anterior Nidopallium	Motor	ALAN = Night (*p* = 0.219)	−1.23	ALAN = Night (*p* = 0.414)	−0.82
		ALAN = Day (*p* = 0.801)	−0.25	ALAN = Day (*p* = 0.982)	0.02
Area Para-hippocampalis		ALAN ≠ Night**(*p* = 0.026)**	−2.23	ALAN = Night (*p* = 0.090)	−1.70
		ALAN = Day (*p* = 0.712)	−0.37	ALAN ≠ Day**(*p* = 0.005)**	−2.79
Anterior Striatum	Motor	ALAN = Night (*p* = 0.749)	−0.32	ALAN = Night (*p* = 0.608)	0.514
		ALAN = Day (*p* = 0.750)	0.32	ALAN ≠ Day**(*p* = 0.017)**	2.39
Caudal Striatum		ALAN = Night (*p* = 0.053)	−1.94	ALAN ≠ Night**(*p* = 0.031)**	−2.15
		ALAN = Day (*p* = 0.129)	−1.52	ALAN = Day (*p* = 0.325)	−0.99
Dorsal Lateral Nidopallium	Motor	ALAN = Night (*p* = 0.401)	0.84	ALAN = Night (*p* = 0.943)	0.07
		ALAN = Day (*p* = 0.362)	0.91	ALAN = Day (*p* = 0.436)	0.78
Entopallium		ALAN ≠ Night**(*p* < 0.001)**	−137.6	ALAN ≠ Night**(*p* < 0.0001)**	−4.79
		ALAN ≠ Day**(*p* < 0.001)**	−195.1	ALAN ≠ Day**(*p* = 0.014)**	−2.46
The core of the Entopallium	Visual	ALAN = Night (*p* = 0.010)	1.65	ALAN ≠ Night**(*p* = 0.038)**	2.08
		ALAN = Day (*p* = 0.058)	1.90	ALAN = Day (*p* = 0.438)	0.78
Hippocampus		ALAN = Night (*p* = 0.251)	1.15	ALAN ≠ Night**(*p* = 0.040)**	2.06
		ALAN = Day (*p* = 0.953)	−0.06	ALAN = Day (*p* = 0.791)	0.27
Lateral Int Arcopallium	Motor	ALAN = Night (*p* = 0.983)	−0.02	ALAN = Night (*p* = 0.394)	0.85
		ALAN ≠ Day**(*p* = 0.030)**	−2.17	ALAN = Day (*p* = 0.448)	−0.76
Lateral Ventral Mesopallium		ALAN ≠ Night**(*p* = 0.001)**	−3.24	ALAN ≠ Night**(*p* = 0.002)**	−3.05
		ALAN = Day (*p* = 0.109)	−1.60	ALAN = Day (*p* = 0.554)	−0.59
Medial Dorsal Mesopallium		ALAN ≠ Night**(*p* = 0.014)**	−2.47	ALAN ≠ Night**(*p* = 0.001)**	−3.33
		ALAN = Day (*p* = 0.206)	−1.27	ALAN ≠ Day**(*p* < 0.0001)**	−3.98
Ventral Mesopallium adjacent to the Basorostral Nucleus	Motor	ALAN = Night (*p* = 0.746)	−0.32	ALAN = Night (*p* = 0.231)	−1.20
		ALAN = Day (*p* = 0.667)	0.43	ALAN ≠ Day**(*p* < 0.0001)**	2.87
Ventral Mesopallium adjacent to the Core of the Entopallium	Visual	ALAN ≠ Night**(*p* = 0.014)**	−2.47	ALAN = Night (*p* = 0.175)	−1.36
		ALAN = Day (*p* = 0.879)	−0.15	ALAN = Day (*p* = 0.590)	0.54
Nidopallium adjacent to the Basorostral Nucleus	Motor	ALAN = Night (*p* = 0.994)	−0.01	ALAN = Night (*p* = 0.671)	−0.42
		ALAN = Day (*p* = 0.180)	1.34	ALAN ≠ Day**(*p* = 0.001)**	3.214
Nidopallium Caudolateral	Motor	ALAN = Night (*p* = 0.462)	−0.74	ALAN ≠ Night**(*p* = 0.011)**	−2.55
		ALAN = Day (*p* = 0.082)	−1.74	ALAN ≠ Day**(*p* = 0.005)**	−2.81
Nidopallium adjacent to the Core of the Entopallium	Visual	ALAN = Night (*p* = 0.716)	−0.36	ALAN ≠ Night**(*p* = 0.019)**	−2.34
		ALAN = Day (*p* = 0.682)	−0.41	ALAN = Day (*p* = 0.411)	0.82
Posterior Dorsal Mesopallium	Visual	ALAN = Night (*p* = 0.149)	−1.44	ALAN = Night (*p* = 0.173)	−1.36
		ALAN = Day (*p* = 0.153)	−1.43	ALAN = Day (*p* = 0.205)	−1.27
Posterior Hyperpallium	Visual	ALAN ≠ Night**(*p* = 0.009)**	−2.60	ALAN = Night (*p* = 0.079)	−1.76
		ALAN ≠ Day**(*p* = 0.017)**	−2.39	ALAN = Day (*p* = 0.072)	−1.80
Posterior Lateral Ventral Mesopallium	Motor	ALAN = Night (*p* = 0.339)	−0.96	ALAN = Night (*p* = 0.363)	−0.91
		ALAN = Day (*p* = 0.297)	−1.04	ALAN = Day (*p* = 0.203)	−1.27
Septum		ALAN = Night (*p* = 0.821)	−0.23	ALAN ≠ Night**(*p* = 0.039)**	2.06
		ALAN = Day (*p* = 0.976)	−0.03	ALAN = Day (*p* = 0.051)	1.95
Striatum adjacent to the Core of the Entopallium	Visual	ALAN ≠ Night**(*p* < 0.0001)**	−215.8	ALAN = Night (*p* = 0.608)	−0.51
		ALAN ≠ Day**(*p* < 0.0001)**	−207.5	ALAN = Day (*p* = 0.326)	0.98

Bold values are significant.

To determine if the expression was based on activity, we reanalyzed expression with birds separated into only two groups of active (*n* = 7) or inactive (*n* = 6; total minutes of activity <1 min) 90 min before perfusion. Active birds included the control day group and non-active included the control night, with the ALAN group split between the two, based on activity. There was no significant difference in cFos or ZENK expression overall between active and non-active birds (cFos: *z* = 1.18, *p* = 0.24, ZENK: *z* = 1.70, *p* = 0.09). Additionally, there was no significant difference between active and non-active birds in the whole motor (cFos: *z* = 1.28, *p* = 0.20, ZENK: *z* = 1.81, *p* = 0.07) or visual (cFos: z = 0.65, *p* = 0.51, ZENK: *z* = 1.37, *p* = 0.17) pathways.

In the visual pathway, the ALAN group showed significantly higher cFos expression in the striatum adjacent to the core of the entopallium, posterior hyperpallium (Figure 2B), and ventral mesopallium adjacent to the core of the entopallium than the control night group, and significantly higher ZENK expression in the nidopallium adjacent to the core of the entopallium but lower in the core of the entopallium. The ALAN group also showed significantly higher cFos expression than the control day group in the striatum adjacent to the core of the entopallium and posterior hyperpallium areas (Table 1).

**Figure 3:**
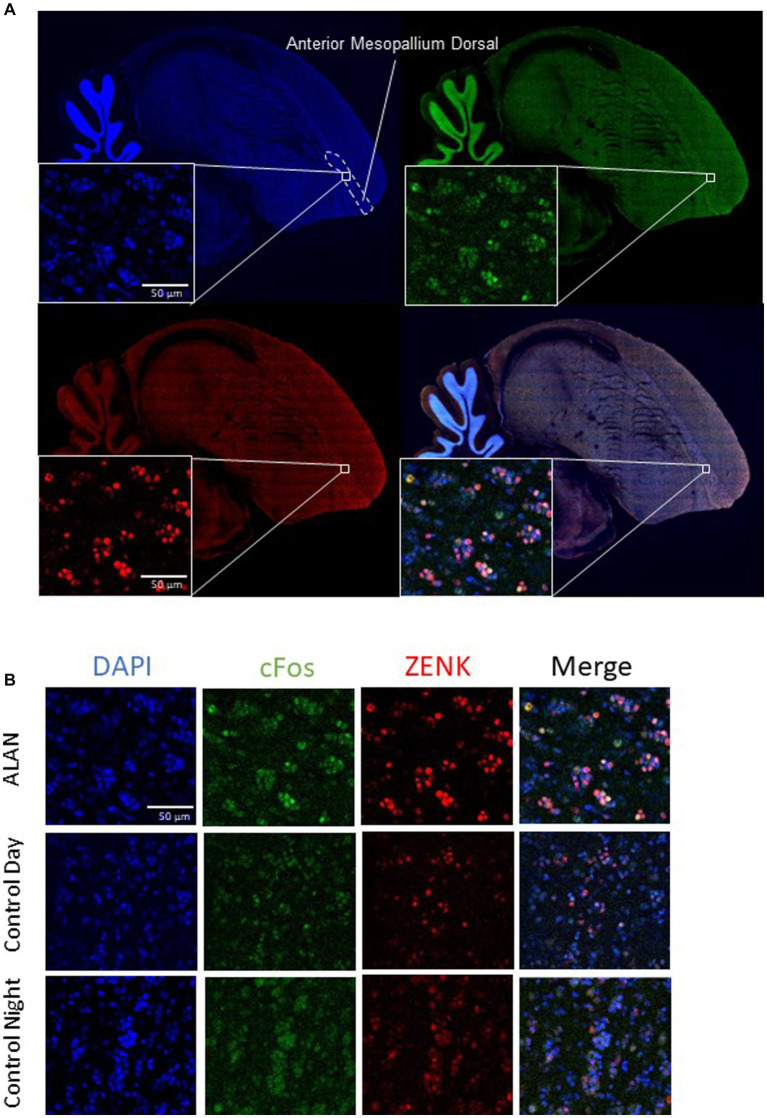
Brain slices with cFos and ZENK staining in the anterior mesopallium dorsal. **(A)** A sagittal slice of a representative zebra finch brain 1 mm from the center, showing the anterior mesopallium dorsal. Blue is DAPI, green is cFos, and red is ZENK expression. **(B)** Images from the anterior mesopallium dorsal of cFos, ZENK, and the overlay of both with DAPI for a bird exposed to ALAN, a bird collected during the day (control day), and a bird collected at night (control night).

In the motor pathway, the ALAN group showed significantly higher cFos expression than the control night group in the anterior mesopallium dorsal (Figure 2B) and anterior mesopallium ventral regions and significantly higher ZENK expression in the anterior mesopallium dorsal and nidopallium caudolateral regions (Figure 3). The ALAN group also had significantly higher levels of cFos expression compared to the control day group in the anterior mesopallium dorsal and lateral int arcopallium and higher ZENK expression in the nidopallium caudolateral. However, the ALAN group had significantly lower ZENK expression in the anterior striatum, nidopallium adjacent to the basorostral nucleus, and ventral mesopallium adjacent to the basorostral nucleus (Table 1). There was no significant difference between active and non-active birds in the anterior mesopallium dorsal, nidopallium caudolateral, or nidopallium adjacent to the basorostral nucleus.

The ALAN group also showed higher cFos expression in the area parahippocampalis, medial dorsal mesopallium, entopallium (Figure 2B), and lateral ventral mesopallium and higher expression of ZENK in the caudal striatum, medial dorsal mesopallium (Figure 2C), entopallium (Figure 2C), and lateral ventral mesopallium as compared to the control night group, but lower levels of ZENK expression in the hippocampus (Figure 2C) and septum. The ALAN group also showed higher levels of cFos expression in the entopallium and higher ZENK expression in the area para-hippocampalis, medial dorsal mesopallium, and entopallium as compared to the control day group (Table 1).

## Discussion

Although ALAN is a pervasive pollutant, the neuronal response remains unclear. We imaged IEG expression of 24 brain regions during the day, night, and ALAN exposure in birds and found various regions were significantly differentially activated among the treatment groups. Overall, ALAN-treated birds were more like control-day birds in total IEG expression. However, six brain regions differed among all three treatment groups: anterior mesopallium dorsal, entopallium, medial dorsal mesopallium, posterior hyperpallium, nidopallium caudolateral, and striatum adjacent to the core of the entopallium.

### Vision

In the visual pathway, control night birds (LD sacrificed during the night) were significantly different from control day (LD sacrificed during the day) and ALAN birds (LLdim sacrificed during the night). These large differences are to be expected as LD control night birds were inactive. However, we still found two areas had significantly stronger cFos expression for ALAN birds than both control groups: posterior hyperpallium and striatum adjacent to the core of the entopallium. ALAN was a novel visual stimulus for the birds, likely employing a visual neuronal response.

The entopallium, the most prominent area to emerge, was significantly different from both controls and both IEGs. The entopallium is involved in visual pattern recognition (Watanabe et al., 2008, 2011). Surprisingly, we found that birds exposed to ALAN had different IEG expression in visual pathways compared to day controls. We see that even very dim levels of ALAN (around 1.5 lux) elicit a clear response in recognizing this visual input.

### Movement

Out of the seven regions of the motor pathway analyzed, ALAN birds were significantly different from the day controls in either cFos or ZENK in six of them. However, when accounting for activity, the ALAN group remained significantly different with increased expression in the anterior mesopallium dorsal and nidopallium caudolateral and decreased in the nidopallium adjacent to the basorostral nucleus. Although the nidopallium caudolateral has additional functions, the anterior mesopallium dorsal and nidopallium adjacent to the basorostral nucleus are differentially activated under ALAN and not associated with hopping. These areas may be picking up movement we did not track, such as head turns and flapping wings, or associated with other functions we are unaware of.

### Memory and learning

We found birds exposed to ALAN were significantly different from both controls in areas associated with learning and memory. The ALAN group had significantly higher IEG expression than the day and night controls in the area para-hippocampalis and medial dorsal mesopallium, which are involved in spatial and object recognition and associative learning, respectively, (He et al., 2010; Damphousse et al., 2022). The ALAN birds also had significantly lower IEG expression than the night controls in the hippocampus, which is involved in spatial memory and learning (Bingman et al., 1990; Mayer et al., 2013). Dim ALAN dampens behavioral measures of learning and memory which have also been correlated with structural alterations in the hippocampus (Taufique et al., 2018, 2019; Liu et al., 2022). Lower nocturnal IEG expression in the hippocampus may partially explain why dim ALAN suppresses gene expression in the hippocampus (Taufique et al., 2018, 2019). It is believed that sleeping activates the hippocampus for memory consolidation (Klinzing et al., 2019). Indeed, we see higher IEG expression in our control night birds than day. A nocturnal suppression of hippocampal activity may impair memory consolidation and learning under ALAN.

ALAN treatment birds had significantly higher IEG expression in the nidopallium caudolateral than either of the controls. This aligns with previous research that has found dim ALAN alters the neuroarchitecture of the nidopallium caudolateral, the avian equivalent of the prefrontal cortex (Gunturkun, 2005; Gunturkun and Bugnyar, 2016; Taufique et al., 2019). The nidopallium caudolateral has been implemented in mimicking prefrontal area structures by having the same receptor architecture as the Brodmann Area 10 in humans, which is involved in many processes including reward and conflict, working memory, and pain (Herold et al., 2011; Peng et al., 2018). IEG activation in areas associated with memory support previous findings that ALAN impairs learning and memory (Liu et al., 2022). Additionally, the avian nidopallium caudolateral along with the entopallium have been shown to display attentional mechanisms (Johnston et al., 2017), implying an alert state in our ALAN exposed birds.

### Pain processing

Another association to emerge was pain processing. Dim ALAN has been shown to alter pain reception in mice (Bumgarner et al., 2020). ALAN treatment birds had significantly higher activity in the caudal striatum from night controls and significantly higher activity in the nidopallium caudolateral from both controls. Although not much is known about the avian caudal striatum, this area is related to anxiety and pain in mice (Jin et al., 2020). Additionally, the nidopallium caudolateral has been associated with the Brodmann Area 10 in humans, also involved in pain reception (Herold et al., 2011; Peng et al., 2018).

### Hormone regulation

Birds under ALAN had significantly decreased IEG expression compared to the control night birds in the septum and hippocampus, which directly regulate hormones leading to downstream physiological changes (Carreras et al., 1984). The hippocampal-septal pathway regulates hormones involved in stress and immune function including; corticotropin-releasing hormone (Vale et al., 1981; Nagarajan et al., 2017), thyrotropin-releasing hormone (Fabris et al., 1995; Ballard et al., 1996), and corticosterone (Garces et al., 1968; Nyakas et al., 1979).

Our results show that ALAN typically increases IEG expression in differentially activated areas compared to both controls. However, reduction of ZENK expression in the septum and hippocampus implies reduced neuronal activation in co-regulated functions—such as hormonal control. This is supported by previous research that ALAN alters hormone production (Ouyang et al., 2018; Moaraf et al., 2020).

In summary, through fine analyses of IEG expression, we found that initial ALAN exposure activates brain areas involved in vision, movement, learning and memory, pain processing, and hormone regulation, which may be differentially regulated under prolonged sleep loss or long-term exposure to ALAN. Additionally, first time exposure to ALAN at a different time in the night may produce differential responses from those we observed. Although ALAN may not be eliciting changes through circadian regulation, we still see substantial responses across brain areas that warrant further study. ALAN creates a unique brain state that is significantly different from day or nighttime brain activity. Dim light creates a novel environment, different from birds active in the day or sleeping at night, which produced widespread differential brain activity.

## Data availability statement

The datasets presented in this study can be found in online repositories. The names of the repository/repositories and accession number(s) can be found at: https://datadryad.org/stash/share/uIyGiK3KEm3Cng_fNAvg9m5pFE65ZJyG4kFOpBO5W3M.

## Ethics statement

The animal study was reviewed and approved by National Institute of Health Ethical Use of Animals.

## Author contributions

CH, VA, and JO designed the experiments. CH, NC, and AC conducted the experiment and completed data analysis. SP and JO oversaw the project, provided training, and reviewed analyses. JO, NC, and AC provided funding. All authors contributed to and reviewed the writing.

## Funding

JO is supported by NIH National Institutes of Health R15 ES030548. NC and AC were supported by the Nevada Undergraduate Research Award and AC by the NEXUS award through the Undergraduate Research Opportunity Program. Research reported in this publication used the Cellular and Molecular Imaging Core facility supported by the National Institute of General Medical Sciences of the National Institutes of Health under grant number P20 GM103650.

## Conflict of interest

The authors declare that the research was conducted in the absence of any commercial or financial relationships that could be construed as a potential conflict of interest.

## Publisher’s note

All claims expressed in this article are solely those of the authors and do not necessarily represent those of their affiliated organizations, or those of the publisher, the editors and the reviewers. Any product that may be evaluated in this article, or claim that may be made by its manufacturer, is not guaranteed or endorsed by the publisher.
