# Supplementary material for: Dim artificial light at night alters immediate early gene expression throughout the avian brain
Source: Front Neurosci. 2023 Jul 4;17:1194996. doi: 10.3389/fnins.2023.1194996 (PMC10352805; doi:10.3389/fnins.2023.1194996)
Supplement: Supplementary file 1 [file Data_Sheet_1.docx]

Supplementary Materials for “**Dim artificial light at night alters immediate early gene expression throughout the avian brain**”

Cassandra K. Hui, Nadya Chen, Arunima Chakraborty, Valentina Alaasam, Simon Pieraut, Jenny Q. Ouyang

**Abbreviations**

AH = Anterior Hyperpallium

AMD = Anterior Mesopallium Dorsal

AMV = Anterior Mesopallium Ventral

AN = Anterior Nidopallium

APH = Area Parahippocampalis

ASt = Anterior Striatum

CSt = Caudal Striatum

DLN = Dorsal Lateral Nidopallium

E = Entopallium

Eco = Core of the Entopallium

HP = Hippocampus

LAi = Lateral Intermediate Arcopallium

MLV = Lateral Ventral Mesopallium

MMD = Medial Dorsal Mesopallium

MVb = Ventral Mesopallium adjacent to the Basorostral Nucleus

MVe = Ventral Mesopallium adjacent to the Eco

Nb = Nidopallium adjacent to Basorostral Nucleus

NCL = Nidopallium Caudolateral

NE = Nidopallium adjacent to the Eco

PH = Posterior Hyperpallium

PLMV = Posterior Lateral Ventral Mesopallium

PMD = Posterior Dorsal Mesopallium

S = Septum

StE = Striatum adjacent to Eco


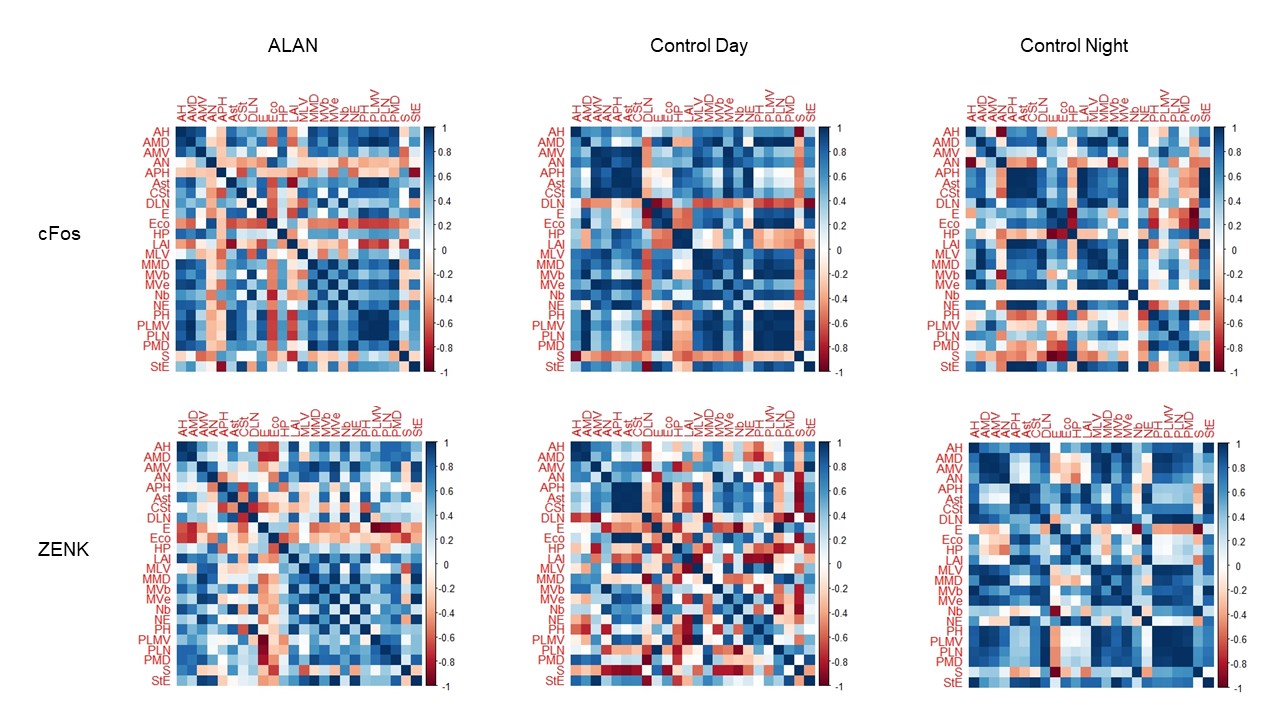


**Figure S1. Correlation matrices of brain regions for Birds exposed to artificial light at night, control day, and control night birds.** A correlation matrix of all brain regions analyzed for birds exposed to artificial light at night, control day, and control night birds. Brain regions listed in alphabetical order of abbreviations. Colors represent strengths of correlations, red= more negative, blue= more positive (see legend).


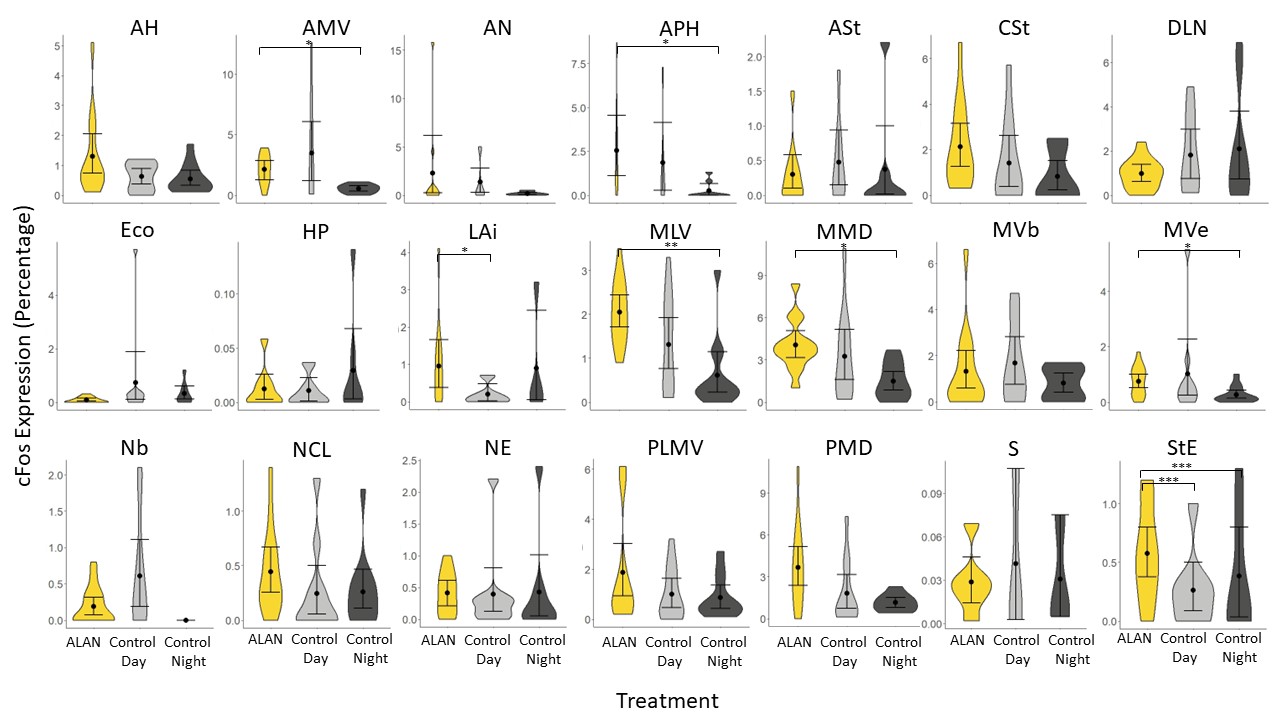


**Figure S2. Immediate early gene expression of cFos throughout the brain for birds exposed to ALAN and for control birds collected during subjective day and night.** cFos expression (percentage) comparing birds exposed to ALAN to control day and control night groups in the AH, AMV, AN, APH, Ast, CSt, DLN, Eco, HP, Lai, MLV, MMD, MVb, MVe, Nb, NCL, NE, PLMV, PMD, S, StE. Shown are means ± 1 SE. Significance stars: ‘*’ *p* < 0.05, ‘**’ *p* < 0.01, ‘***’ *p* < 0.001.


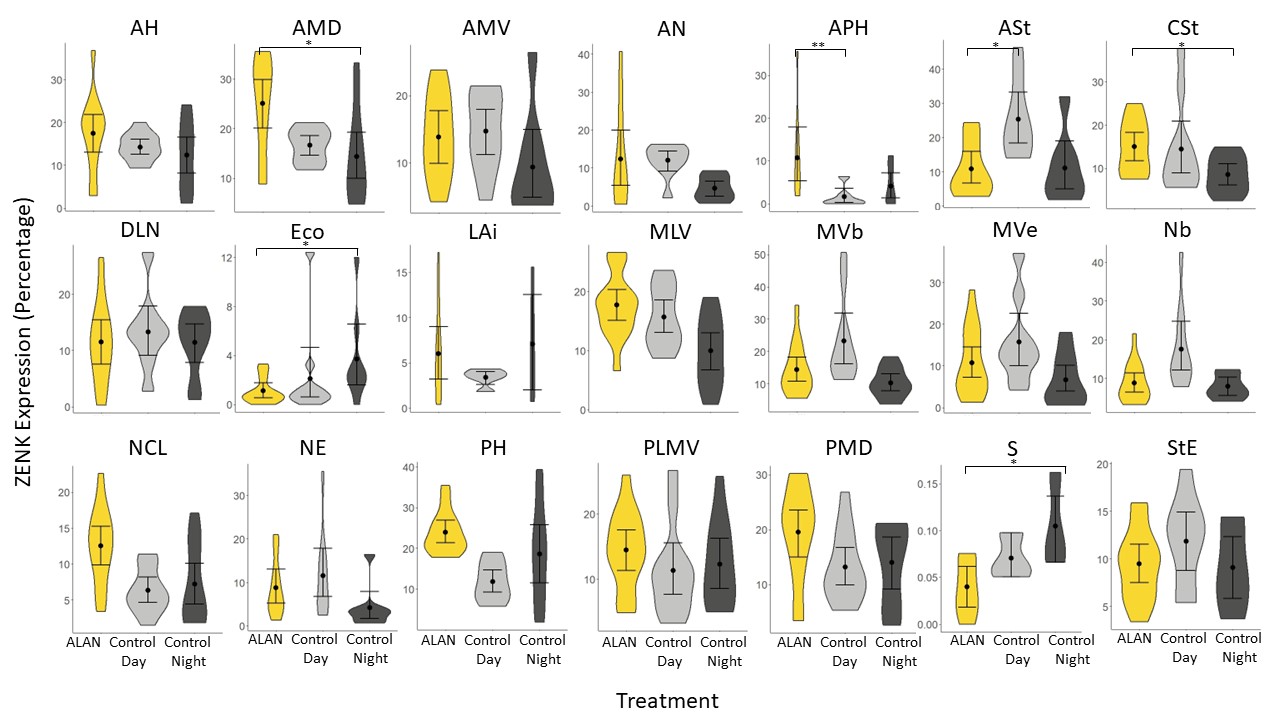


**Figure S3. Immediate early gene expression of ZENK throughout the brain for birds exposed to ALAN, and control birds collected during subjective day and night.** ZENK expression (percentage) comparing birds exposed to ALAN to control day and control night groups in the AH, AMD, AMV, AN, APH, Ast, CSt, DLN, Eco, Lai, MLV, MVb, MVe, Nb, NCL, NE, PH, PLMV, PMD, S, StE. Shown are means ± 1 SE. Significance stars: ‘*’ *p* < 0.05, ‘**’ *p* < 0.01, ‘***’ *p* < 0.001.
